# Supplementary material for: High Proportions of Radiation-Resistant Strains in Culturable Bacteria from the Taklimakan Desert
Source: Biology (Basel). 2022 Mar 24;11(4):501. doi: 10.3390/biology11040501 (PMC9030528; doi:10.3390/biology11040501)
Supplement: Supplementary file 1 [file biology-11-00501-s001.zip › biology-1616095-supplementary.pdf]

Supplementary Figures

(A)

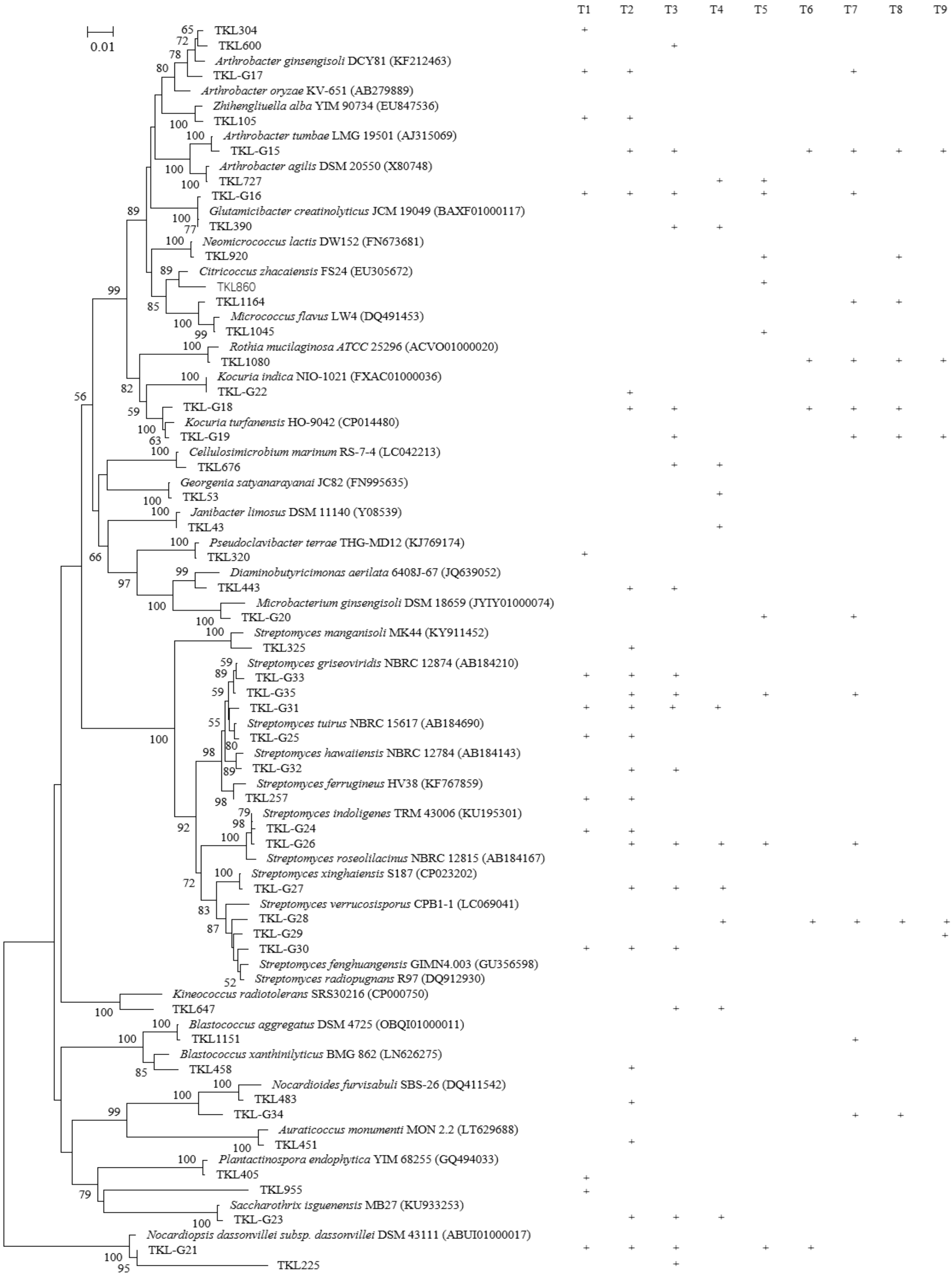

(B)

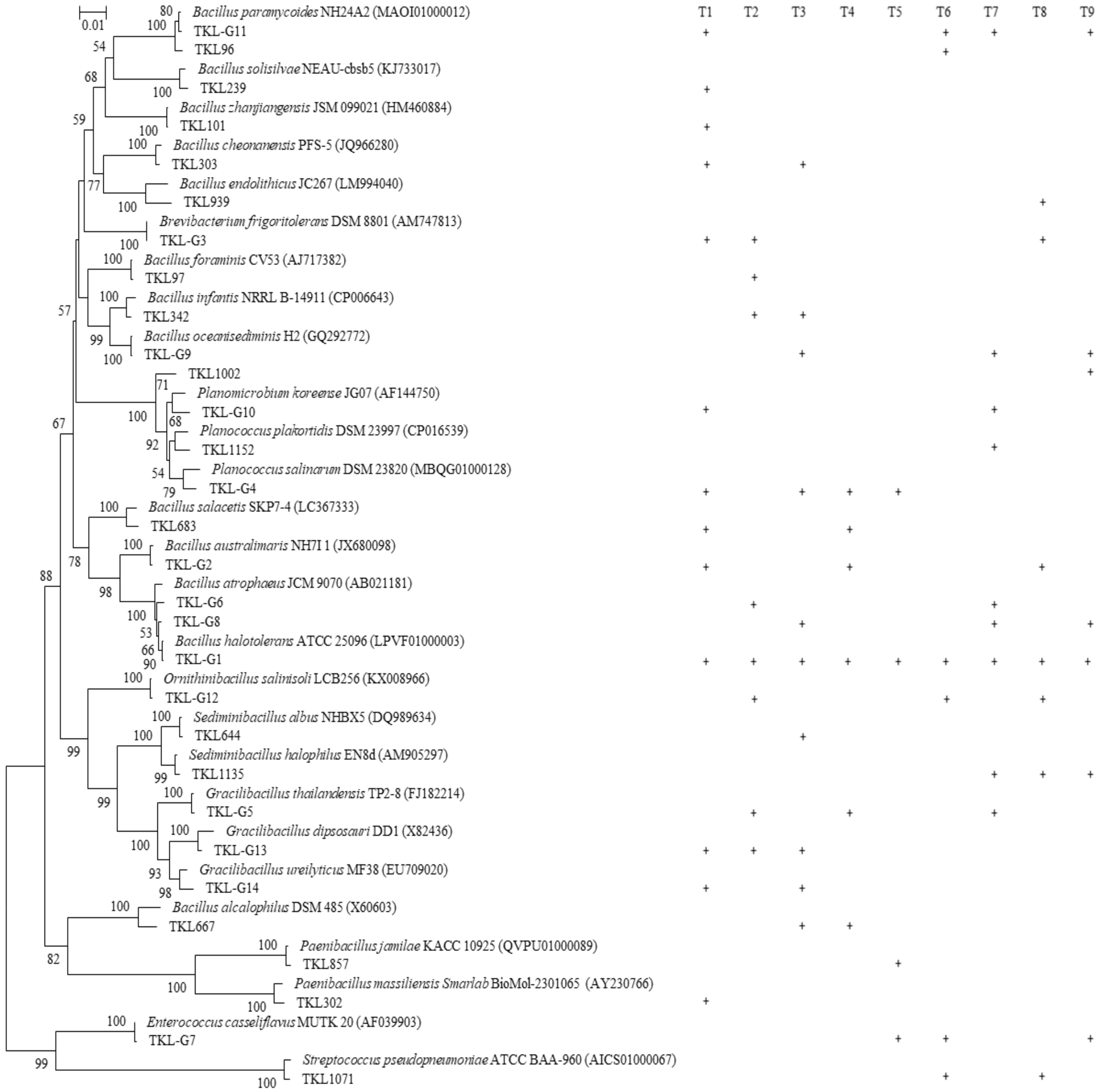

(C)

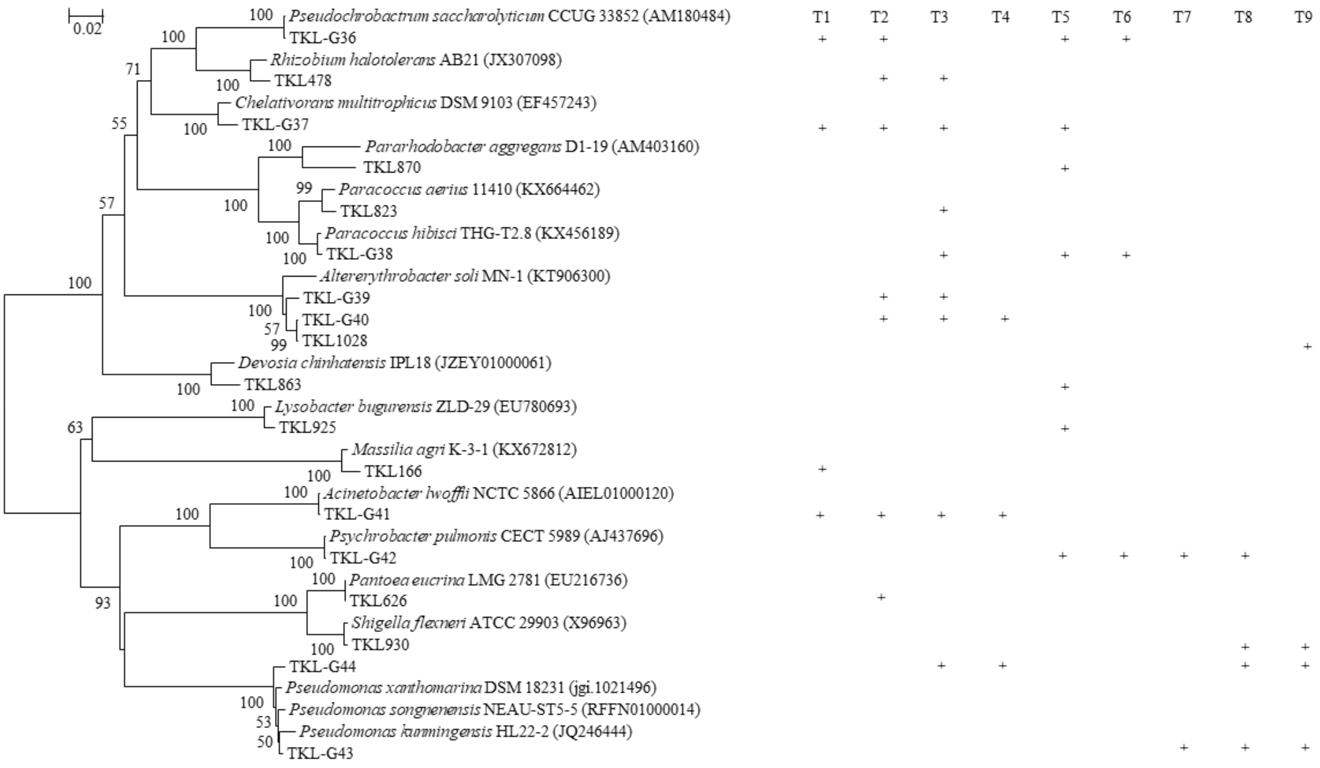

(D)

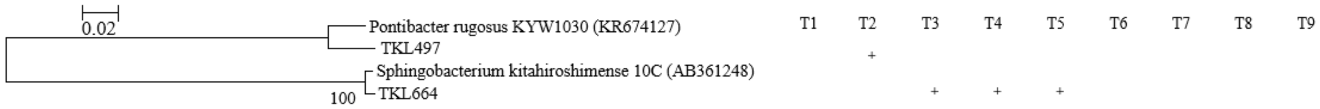

Figure S1. Neighbour-joining phylogenetic trees of the phyla of Actinobacteria (A), Firmicutes (B), Proteobacteria (C), and Bacteroidetes (D) based on the 16S rRNA gene sequences and the distribution of the phylotypes. The symbol + refers that the stain was cultured in the sites (T1~T9). The TKL-GN (N = 1~44) represent strain of the group 1 - 44, in which the strains from the same group have the higher similarity than 99% of the 16S rRNA gene sequences (Details in Table S1). The numbers at the nodes indicate the level of bootstrap support based on neighbor-joining tree reconstruction with 1000 resampling; bootstrap values higher than 50% are showed.

Supplementary Table

Table S1. Phylogenetic similarity and color of culturable bacteria in the Taklimakan Desert

Strain No.	Similarity Strain	Accession No.	Similarity/ %	Group	Color
TKL21	*Bacillus tequilensis* KCTC 13622	MW827806	99.86	G1	White
TKL22	*Bacillus tequilensis* KCTC 13622	MW827807	99.93	G1	White
TKL40	*Bacillus subtilis subsp. Stercoris* D7XPN1	MW827816	99.84	G1	White
TKL44	*Bacillus subtilis subsp. Stercoris* D7XPN1	MW827818	99.84	G1	White
TKL45	*Bacillus tequilensis* KCTC 13622	MW827819	99.86	G1	White
TKL75	*Bacillus subtilis subsp. Stercoris* D7XPN1	MW827825	99.20	G1	White
TKL77	*Bacillus subtilis subsp. Stercoris* D7XPN1	MW827826	99.84	G1	White
TKL78	*Bacillus halotolerans* ATCC 25096	MW827827	99.93	G1	White
TKL80	*Bacillus subtilis subsp. Stercoris* D7XPN1	MW827828	99.84	G1	White
TKL86	*Bacillus subtilis subsp. Stercoris* D7XPN1	MW827830	99.92	G1	White
TKL89	*Bacillus subtilis subsp. Stercoris* D7XPN1	MW827831	99.92	G1	White
TKL90	*Bacillus subtilis subsp. Stercoris* D7XPN1	MW827832	99.92	G1	White
TKL98	*Bacillus halotolerans* ATCC 25096	MW827839	99.93	G1	White
TKL104	*Bacillus halotolerans* ATCC 25096	MW827843	99.93	G1	White
TKL106	*Bacillus subtilis subsp. Stercoris* D7XPN1	MW827845	99.92	G1	White
TKL109	*Bacillus subtilis subsp. Stercoris* D7XPN1	MW827846	99.92	G1	White
TKL114	*Bacillus halotolerans* ATCC 25096	MW827847	99.93	G1	White
TKL116	*Bacillus subtilis subsp. Inaquosorum* KCTC 13429	MW827848	100.00	G1	White
TKL117	*Bacillus subtilis subsp. Stercoris* D7XPN1	MW827849	99.92	G1	White
TKL123	*Bacillus tequilensis* KCTC 13622	MW827852	99.86	G1	White
TKL126	*Bacillus subtilis subsp. Stercoris* D7XPN1	MW827853	99.84	G1	White
TKL130	*Bacillus halotolerans* ATCC 25096	MW827854	99.93	G1	White
TKL131	*Bacillus halotolerans* ATCC 25096	MW827855	99.86	G1	White
TKL134	*Bacillus subtilis subsp. Stercoris* D7XPN1	MW827856	99.68	G1	White
TKL135	*Bacillus halotolerans* ATCC 25096	MW827857	99.93	G1	White
TKL139	*Bacillus halotolerans* ATCC 25096	MW827858	100.00	G1	White
TKL142	*Bacillus subtilis subsp. Stercoris* D7XPN1	MW827859	99.92	G1	White
TKL144	*Bacillus subtilis subsp. Stercoris* D7XPN1	MW827860	99.92	G1	White
TKL149	*Bacillus subtilis subsp. Stercoris* D7XPN1	MW827862	99.60	G1	White
TKL212	*Bacillus halotolerans* ATCC 25096	MW827871	99.93	G1	White
TKL220	*Bacillus subtilis subsp. Stercoris* D7XPN1	MW827872	99.92	G1	White
TKL221	*Bacillus subtilis subsp. Stercoris* D7XPN1	MW827873	99.92	G1	White
TKL222	*Bacillus subtilis subsp. Stercoris* D7XPN1	MW827874	99.92	G1	White
TKL228	*Bacillus subtilis subsp. Stercoris* D7XPN1	MW827877	99.92	G1	White
TKL232	*Bacillus halotolerans* ATCC 25096	MW827878	99.93	G1	White
TKL235	*Bacillus halotolerans* ATCC 25096	MW827880	99.93	G1	White
TKL236	*Bacillus halotolerans* ATCC 25096	MW827881	99.93	G1	White
TKL247	*Bacillus subtilis subsp. Inaquosorum* KCTC 13429	MW827886	99.86	G1	White
TKL248	*Bacillus subtilis subsp. Stercoris* D7XPN1	MW827887	99.92	G1	White
TKL250	*Bacillus subtilis subsp. Stercoris* D7XPN1	MW827888	99.92	G1	White
TKL258	*Bacillus subtilis subsp. Stercoris* D7XPN1	MW827890	99.92	G1	White

Strain No.	Similarity Strain	Accession No.	Similarity/ %	Group	Color
TKL261	*Bacillus subtilis subsp. Stercoris* D7XPN1	MW827891	99.92	G1	White
TKL264	*Bacillus tequilensis* KCTC 13622	MW827893	99.93	G1	White
TKL280	*Bacillus halotolerans* ATCC 25096	MW827895	99.93	G1	White
TKL283	*Bacillus halotolerans* ATCC 25096	MW827897	99.93	G1	White
TKL285	*Bacillus halotolerans* ATCC 25096	MW827898	99.93	G1	White
TKL292	*Bacillus subtilis subsp. Stercoris* D7XPN1	MW827900	99.92	G1	White
TKL293	*Bacillus subtilis subsp. Stercoris* D7XPN1	MW827901	99.92	G1	White
TKL294	*Bacillus halotolerans* ATCC 25096	MW827902	99.93	G1	Red
TKL295	*Bacillus subtilis subsp. Stercoris* D7XPN1	MW827903	99.92	G1	White
TKL296	*Bacillus subtilis subsp. Inaquosorum* KCTC 13429	MW827904	100.00	G1	White
TKL298	*Bacillus subtilis subsp. Inaquosorum* KCTC 13429	MW827905	100.00	G1	White
TKL314	*Bacillus subtilis subsp. Inaquosorum* KCTC 13429	MW827912	100.00	G1	White
TKL350	*Bacillus subtilis subsp. Stercoris* D7XPN1	MW827922	99.92	G1	White
TKL355	*Bacillus halotolerans* ATCC 25096	MW827925	99.93	G1	White
TKL361	*Bacillus subtilis subsp. Stercoris* D7XPN1	MW827926	99.92	G1	White
TKL362	*Bacillus tequilensis* KCTC 13622	MW827927	99.86	G1	White
TKL367	*Bacillus halotolerans* ATCC 25096	MW827928	99.93	G1	White
TKL398	*Bacillus subtilis subsp. Stercoris* D7XPN1	MW827933	99.92	G1	White
TKL403	*Bacillus subtilis subsp. Stercoris* D7XPN1	MW827935	99.92	G1	White
TKL407	*Bacillus subtilis subsp. Stercoris* D7XPN1	MW827937	99.20	G1	White
TKL412	*Bacillus subtilis subsp. Stercoris* D7XPN1	MW827938	99.68	G1	White
TKL437	*Bacillus subtilis subsp. Stercoris* D7XPN1	MW827942	99.60	G1	White
TKL459	*Bacillus subtilis subsp. Stercoris* D7XPN1	MW827952	99.92	G1	White
TKL468	*Bacillus subtilis subsp. Stercoris* D7XPN1	MW827954	99.92	G1	White
TKL481	*Bacillus subtilis subsp. Spizizenii* NRRL B-23049	MW827956	99.93	G1	White
TKL585	*Bacillus halotolerans* ATCC 25096	MW827984	99.93	G1	White
TKL588	*Bacillus subtilis subsp. Inaquosorum* KCTC 13429	MW827985	99.93	G1	White
TKL607	*Bacillus tequilensis* KCTC 13622	MW827995	99.93	G1	White
TKL609	*Bacillus atrophaeus* JCM 9070	MW827997	99.93	G1	White
TKL677	*Bacillus subtilis subsp. Stercoris* D7XPN1	MW828023	99.92	G1	White
TKL637	*Bacillus subtilis subsp. Stercoris* D7XPN1	MW828005	99.92	G1	White
TKL657	*Bacillus subtilis subsp. Stercoris* D7XPN1	MW828016	99.92	G1	White
TKL658	*Bacillus subtilis subsp. Stercoris* D7XPN1	MW828017	99.92	G1	White
TKL674	*Bacillus halotolerans* ATCC 25096	MW828020	99.93	G1	White
TKL680	*Bacillus atrophaeus* JCM 9070	MW828024	99.85	G1	White
TKL689	*Bacillus halotolerans* ATCC 25096	MW828027	99.93	G1	White
TKL692	*Bacillus subtilis subsp. Stercoris* D7XPN1	MW828029	99.92	G1	White
TKL819	*Bacillus subtilis subsp. Stercoris* D7XPN1	MW828034	99.92	G1	White
TKL873	*Bacillus subtilis subsp. Stercoris* D7XPN1	MW828046	99.92	G1	White
TKL875	*Bacillus subtilis subsp. Stercoris* D7XPN1	MW828047	99.92	G1	White
TKL889	*Bacillus subtilis subsp. Inaquosorum* KCTC 13429	MW828050	99.86	G1	White
TKL916	*Bacillus subtilis subsp. Stercoris* D7XPN1	MW828055	99.68	G1	White
TKL918	*Bacillus subtilis subsp. Stercoris* D7XPN1	MW828057	99.68	G1	White

Strain No.	Similarity Strain	Accession No.	Similarity/ %	Group	Color
TKL927	*Bacillus subtilis subsp. Stercoris* D7XPN1	MW828060	99.52	G1	White
TKL938	*Bacillus subtilis subsp. Inaquosorum* KCTC 13429	MW828064	99.86	G1	White
TKL1018	*Bacillus subtilis subsp. Stercoris* D7XPN1	MW828080	99.92	G1	White
TKL1025	*Bacillus subtilis subsp. Stercoris* D7XPN1	MW828084	99.92	G1	White
TKL1044	*Bacillus subtilis subsp. Inaquosorum* KCTC 13429	MW828090	100.00	G1	White
TKL1046	*Bacillus subtilis subsp. Stercoris* D7XPN1	MW828092	100.00	G1	White
TKL1052	*Bacillus subtilis subsp. Stercoris* D7XPN1	MW828095	99.92	G1	White
TKL1053	*Bacillus subtilis subsp. Stercoris* D7XPN1	MW828096	99.92	G1	White
TKL1058	*Bacillus subtilis subsp. Stercoris* D7XPN1	MW828100	99.76	G1	White
TKL1059	*Bacillus subtilis subsp. Stercoris* D7XPN1	MW828101	99.52	G1	White
TKL1088	*Bacillus subtilis subsp. Stercoris* D7XPN1	MW828110	99.84	G1	White
TKL1120	*Bacillus subtilis subsp. Stercoris* D7XPN1	MW828122	99.68	G1	White
TKL1122	*Bacillus tequilensis* KCTC 13622	MW828124	99.72	G1	White
TKL1125	*Bacillus subtilis subsp. Stercoris* D7XPN1	MW828125	99.68	G1	White
TKL1128	*Bacillus subtilis subsp. Stercoris* D7XPN1	MW828127	99.76	G1	White
TKL38	*Bacillus australimaris* NH7I_1	MW827815	99.93	G2	White
TKL318	*Bacillus altitudinis* 41KF2b	MW827914	100.00	G2	White
TKL942	*Bacillus pumilus* ATCC 7061	MW828066	99.79	G2	White
TKL512	*Brevibacterium frigoritolerans* DSM 8801	MW827967	100.00	G3	Red
TKL321	*Brevibacterium frigoritolerans* DSM 8801	MW827916	99.86	G3	White
TKL640	*Planomicrobium koreense* JG07	MW828006	99.06	G4	White
TKL27	*Planococcus salinarum* DSM 23820	MW827811	98.51	G4	White
TKL19	*Gracilibacillus thailandensis* TP2-8	MW827804	98.90	G5	Pink
TKL51	*Gracilibacillus saliphilus* YIM 91119	MW827822	100.00	G5	Red
TKL442	*Enterococcus casseliflavus* MUTK 20	MW827944	99.93	G6	White
TKL1155	*Staphylococcus hominis subsp. Novobiosepticus* GTC 1228	MW828139	99.51	G6	White
TKL48	*Bacillus velezensis* CR-502	MW827820	99.93	G7	White
TKL1097	*Bacillus velezensis* CR-502	MW828114	99.78	G7	White
TKL23	*Bacillus velezensis* CR-502	MW827808	99.57	G7	White
TKL1015	*Bacillus tequilensis* KCTC 13622	MW828078	99.93	G8	White
TKL1016	*Bacillus subtilis subsp. Stercoris* D7XPN1	MW828079	99.84	G8	White
TKL1163	*Bacillus oceanisediminis* H2	MW828143	99.93	G9	White
TKL1012	*Bacillus oceanisediminis* H2	MW828077	99.93	G9	White
TKL650	*Bacillus oceanisediminis* H2	MW828012	99.64	G9	White
TKL25	*Planomicrobium koreense* JG07	MW827809	98.86	G10	Yellow
TKL1160	*Planomicrobium koreense* JG07	MW828142	98.60	G10	White
TKL9	*Bacillus thuringiensis* ATCC 10792	MW827799	99.93	G11	White
TKL15	*Bacillus thuringiensis* ATCC 10792	MW827800	99.86	G11	White
TKL94	*Bacillus thuringiensis* ATCC 10792	MW827835	99.93	G11	White
TKL95	*Bacillus thuringiensis* ATCC 10792	MW827836	99.86	G11	White
TKL103	*Bacillus paramycoides* NH24A2	MW827842	99.86	G11	White
TKL1026	*Bacillus paramycoides* NH24A2	MW828085	99.79	G11	White
TKL1090	*Bacillus cereus* ATCC 14579	MW828111	99.67	G11	White

Strain No.	Similarity Strain	Accession No.	Similarity/ %	Group	Color
TKL1095	*Bacillus paramycoides* NH24A2	MW828113	99.79	G11	White
TKL1105	*Bacillus paramycoides* NH24A2	MW828116	99.38	G11	White
TKL1121	*Bacillus paramycoides* NH24A2	MW828123	99.72	G11	White
TKL434	*Ornithinibacillus salinisoli* LCB256	MW827941	99.86	G12	White
TKL931	*Ornithinibacillus salinisoli* LCB256	MW828062	99.79	G12	White
TKL1067	*Oceanobacillus kimchii* X50	MW828104	99.86	G12	White
TKL242	*Gracilibacillus dipsosauri* DD1	MW827884	99.37	G13	White
TKL244	*Gracilibacillus dipsosauri* DD1	MW827885	99.23	G13	White
TKL190	*Gracilibacillus ureilyticus* MF38	MW827869	99.23	G14	White
TKL156	*Gracilibacillus ureilyticus* MF38	MW827864	98.88	G14	White
TKL97	*Bacillus foraminis* CV53	MW827838	99.93	none	White
TKL101	*Bacillus zhanjiangensis* JSM 099021	MW827841	99.93	none	White
TKL939	*Bacillus endolithicus* JC267	MW828065	98.28	none	White
TKL303	*Bacillus cheonanensis* PFS-5	MW827907	99.79	none	White
TKL1071	*Streptococcus pseudopneumoniae* ATCC BAA-960	MW828106	99.51	none	White
TKL1002	*Planococcus plakortidis* DSM 23997	MW828076	98.09	none	White
TKL1152	*Planococcus citreus* DSM 20549	MW828138	99.64	none	Red
TKL342	*Bacillus infantis* NRRL B-14911	MW827920	99.58	none	White
TKL683	*Bacillus salacetis* SKP7-4	MW828025	99.06	none	White
TKL239	*Bacillus solisilvae* NEAU-cbsb5	MW827883	99.35	none	White
TKL96	*Bacillus thuringiensis* ATCC 10792	MW827837	99.86	none	White
TKL644	*Sediminibacillus albus* NHBX5	MW828008	99.93	none	White
TKL1135	*Sediminibacillus halophilus* EN8d	MW828131	99.65	none	White
TKL667	*Bacillus alcalophilus* DSM 485	MW828019	98.76	none	White
TKL302	*Paenibacillus massiliensisSmarlab* BioMol-2301065	MW827906	99.29	none	White
TKL857	*Paenibacillus jamilae* KACC 10925	MW828040	99.65	none	White
TKL523	*Arthrobacter tumbae* LMG 19501	MW827968	98.87	G15	White
TKL550	*Arthrobacter tumbae* LMG 19501	MW827977	99.57	G15	White
TKL594	*Arthrobacter tumbae* LMG 19501	MW827987	99.64	G15	White
TKL648	*Arthrobacter tumbae* LMG 19501	MW828010	99.78	G15	White
TKL824	*Arthrobacter tumbae* LMG 19501	MW828037	99.50	G15	White
TKL946	*Arthrobacter tumbae* LMG 19501	MW828067	99.50	G15	White
TKL1054	*Arthrobacter tumbae* LMG 19501	MW828097	99.64	G15	White
TKL1069	*Arthrobacter tumbae* LMG 19501	MW828105	99.78	G15	White
TKL1099	*Arthrobacter tumbae* LMG 19501	MW828115	99.15	G15	White
TKL1130	*Arthrobacter tumbae* LMG 19501	MW828128	98.57	G15	White
TKL1159	*Arthrobacter tumbae* LMG 19501	MW828141	99.50	G15	White
TKL1167	*Arthrobacter tumbae* LMG 19501	MW828147	99.57	G15	White
TKL309	*Modestobacter roseus* KLBMP 1279	MW827911	99.85	G16	White
TKL390	*Glutamicibacter creatinolyticus* JCM 19049	MW827929	99.85	G16	Red
TKL820	*Glutamicibacter creatinolyticus* JCM 19049	MW828035	99.84	G16	Red
TKL859	*Glutamicibacter creatinolyticus* JCM 19049	MW828041	99.85	G16	White
TKL306	*Arthrobacter oryzae* KV-651	MW827909	98.99	G17	White

Strain No.	Similarity Strain	Accession No.	Similarity/ %	Group	Color
TKL308	*Arthrobacter oryzae* KV-651	MW827910	98.89	G17	White
TKL541	*Arthrobacter ginsengisoli* DCY81	MW827973	99.04	G17	White
TKL1133	*Arthrobacter oryzae* KV-651	MW828129	98.99	G17	White
TKL506	*Kocuria turfanensis* HO-9042	MW827964	99.43	G18	Gold
TKL556	*Kocuria rosea* DSM 20447	MW827979	99.28	G18	Gold
TKL953	*Kocuria turfanensis* HO-9042	MW828070	98.71	G18	Gold
TKL1001	*Kocuria polaris* CMS 76or	MW828075	100.00	G18	Gold
TKL1092	*Kocuria turfanensis* HO-9042	MW828112	99.64	G18	Pink
TKL570	*Kocuria rosea* DSM 20447	MW827982	99.78	G19	Red
TKL1060	*Kocuria salina* Hv14b	MW828102	99.28	G19	Red
TKL1106	*Kocuria salina* Hv14b	MW828117	99.28	G19	Red
TKL737	*Microbacterium ginsengisoli* DSM 18659	MW828031	98.22	G20	White
TKL1144	*Microbacterium ginsengisoli* DSM 18659	MW828134	98.64	G20	White
TKL26	*Nocardiopsis sinuspersici* HM6	MW827810	98.78	G21	White
TKL197	*Nocardiopsis dassonvillei subsp. Dassonvillei* DSM 43111	MW827870	98.64	G21	White
TKL237	*Nocardiopsis dassonvillei subsp. Dassonvillei* DSM 43111	MW827882	98.85	G21	White
TKL338	*Nocardiopsis sinuspersici* HM6	MW827919	98.78	G21	White
TKL400	*Nocardiopsis sinuspersici* HM6	MW827934	98.78	G21	White
TKL491	*Saccharothrix isguenensis* MB27	MW827960	99.13	G21	White
TKL656	*Nocardiopsis sinuspersici* HM6	MW828015	98.78	G21	White
TKL1138	*Nocardiopsis dassonvillei subsp. Dassonvillei* DSM 43111	MW828132	98.51	G21	White
TKL1057	*Kocuria indica* NIO-1021	MW828099	99.93	G22	Gold
TKL1117	*Kocuria indica* NIO-1021	MW828120	99.64	G22	White
TKL262	*Saccharothrix isguenensis* MB27	MW827892	99.27	G23	White
TKL426	*Saccharothrix isguenensis* MB27	MW827940	99.13	G23	White
TKL453	*Saccharothrix isguenensis* MB27	MW827950	99.13	G23	White
TKL485	*Nocardiopsis dassonvillei subsp. Dassonvillei* DSM 43111	MW827958	99.43	G23	White
TKL488	*Saccharothrix isguenensis* MB27	MW827959	99.13	G23	White
TKL649	*Saccharothrix isguenensis* MB27	MW828011	99.17	G23	White
TKL50	*Streptomyces luridus* NBRC 12793	MW827821	99.48	G24	White
TKL91	*Streptomyces roseolilacinus* NBRC 12815	MW827833	99.26	G24	White
TKL99	*Streptomyces luteus* TRM 45540	MW827840	99.78	G25	White
TKL281	*Streptomyces luteus* TRM 45540	MW827896	98.95	G25	White
TKL18	*Streptomyces indoligenes* TRM 43006	MW827803	99.92	G26	White
TKL32	*Streptomyces roseolilacinus* NBRC 12815	MW827813	99.26	G26	White
TKL605	*Streptomyces roseolilacinus* NBRC 12815	MW827993	99.49	G26	White
TKL622	*Streptomyces roseolilacinus* NBRC 12815	MW828000	99.41	G26	White
TKL630	*Streptomyces roseolilacinus* NBRC 12815	MW828003	99.28	G26	White
TKL911	*Streptomyces roseolilacinus* NBRC 12815	MW828053	99.42	G26	White
TKL1166	*Streptomyces roseolilacinus* NBRC 12815	MW828146	99.42	G26	Navy Blue
TKL17	*Streptomyces xinghaiensis* S187	MW827802	99.93	G27	White
TKL120	*Streptomyces xinghaiensis* S187	MW827850	99.93	G27	White
TKL265	*Streptomyces xinghaiensis* S187	MW827894	100.00	G27	White

Strain No.	Similarity Strain	Accession No.	Similarity/ %	Group	Color
TKL336	*Streptomyces xinghaiensis* S187	MW827918	99.93	G27	White
TKL422	*Streptomyces xinghaiensis* S187	MW827939	99.93	G27	White
TKL16	*Streptomyces radiopugnans* R97	MW827801	98.60	G28	White
TKL74	*Streptomyces radiopugnans* R97	MW827824	98.52	G28	White
TKL684	*Streptomyces fenghuangensis* GIMN4.003	MW828026	99.01	G28	White
TKL690	*Streptomyces mangrovi* HA11110	MW828028	98.60	G28	White
TKL1047	*Streptomyces fenghuangensis* GIMN4.003	MW828093	98.28	G28	White
TKL1157	*Streptomyces radiopugnans* R97	MW828140	98.43	G28	White
TKL578	*Streptomyces mangrovi* HA11110	MW827983	99.41	G29	White
TKL596	*Streptomyces mangrovi* HA11110	MW827989	99.34	G29	White
TKL1019	*Streptomyces mangrovi* HA11110	MW828081	99.34	G29	White
TKL81	*Streptomyces verrucosisporus* CPB1-1	MW827829	99.18	G30	White
TKL159	*Streptomyces radiopugnans* R97	MW827865	98.97	G30	White
TKL394	*Streptomyces radiopugnans* R97	MW827932	99.11	G30	White
TKL31	*Streptomyces atrovirens* NRRL B-16357	MW827812	99.11	G31	White
TKL122	*Streptomyces pseudogriseolus* NRRL B-3288	MW827851	99.04	G31	Pink
TKL147	*Streptomyces pseudogriseolus* NRRL B-3288	MW827861	99.10	G31	Pink
TKL233	*Streptomyces pseudogriseolus* NRRL B-3288	MW827879	99.04	G31	Pink
TKL538	*Streptomyces griseoviridis* NBRC 12874	MW827972	98.97	G31	White
TKL635	*Streptomyces pseudogriseolus* NRRL B-3288	MW828004	99.04	G31	White
TKL1143	*Streptomyces pseudogriseolus* NRRL B-3288	MW828133	99.14	G31	White
TKL348	*Streptomyces atrovirens* NRRL B-16357	MW827921	99.70	G32	White
TKL351	*Streptomyces atrovirens* NRRL B-16357	MW827923	99.71	G32	Red
TKL450	*Streptomyces atrovirens* NRRL B-16357	MW827947	99.78	G32	Red
TKL511	*Streptomyces hawaiiensis* NBRC 12784	MW827966	99.33	G32	White
TKL567	*Streptomyces atrovirens* NRRL B-16357	MW827981	99.50	G32	White
TKL1146	*Streptomyces atrovirens* NRRL B-16357	MW828136	99.78	G32	White
TKL153	*Streptomyces thinghirensis* DSM 41919	MW827863	99.63	G33	White
TKL227	*Streptomyces thinghirensis* DSM 41919	MW827876	99.42	G33	White
TKL566	*Streptomyces griseoviridis* NBRC 12874	MW827980	98.23	G33	White
TKL968	*Nocardioides terrigena* DS-17	MW828073	99.78	G34	White
TKL1056	*Nocardioides terrigena* DS-17	MW828098	99.78	G34	White
TKL7	*Streptomyces griseoviridis* NBRC 12874	MW827797	98.97	G35	White
TKL171	*Streptomyces griseoviridis* NBRC 12874	MW827867	98.31	G35	White
TKL186	*Streptomyces griseoviridis* NBRC 12874	MW827868	98.97	G35	White
TKL315	*Streptomyces griseoviridis* NBRC 12874	MW827913	98.97	G35	White
TKL352	*Streptomyces griseoviridis* NBRC 12874	MW827924	98.97	G35	White
TKL461	*Streptomyces griseoviridis* NBRC 12874	MW827953	98.98	G35	White
TKL503	*Streptomyces griseoviridis* NBRC 12874	MW827963	98.98	G35	White
TKL531	*Nocardiopsis dassonvillei subsp. Dassonvillei* DSM 43111	MW827969	95.06	G35	White
TKL533	*Streptomyces griseoviridis* NBRC 12874	MW827970	98.98	G35	White
TKL542	*Streptomyces griseoviridis* NBRC 12874	MW827974	98.97	G35	White
TKL548	*Streptomyces pseudogriseolus* NRRL B-3288	MW827975	99.04	G35	White

Strain No.	Similarity Strain	Accession No.	Similarity/ %	Group	Color
TKL549	*Streptomyces griseoviridis* NBRC 12874	MW827976	98.97	G35	White
TKL555	*Streptomyces griseoviridis* NBRC 12874	MW827978	98.98	G35	White
TKL599	*Streptomyces griseoviridis* NBRC 12874	MW827990	98.83	G35	White
TKL608	*Streptomyces griseoviridis* NBRC 12874	MW827996	98.95	G35	White
TKL619	*Streptomyces griseoviridis* NBRC 12874	MW827998	98.98	G35	White
TKL620	*Streptomyces griseoviridis* NBRC 12874	MW827999	98.98	G35	White
TKL751	*Streptomyces griseoviridis* NBRC 12874	MW828032	98.95	G35	White
TKL908	*Streptomyces griseoviridis* NBRC 12874	MW828052	98.38	G35	White
TKL897	*Streptomyces griseoviridis* NBRC 12874	MW828051	98.99	G35	White
TKL913	*Streptomyces griseoviridis* NBRC 12874	MW828054	98.99	G35	White
TKL1134	*Streptomyces griseoviridis* NBRC 12874	MW828130	98.99	G35	White
TKL43	*Janibacter limosus* DSM 11140	MW827817	99.78	none	White
TKL53	*Georgenia satyanarayanai* JC82	MW827823	99.77	none	Red
TKL92	*Streptomyces tuirus* NBRC 15617	MW827834	99.48	none	White
TKL105	*Zhihengliuella alba* YIM 90734	MW827844	99.34	none	White
TKL225	*Nocardiopsis dassonvillei subsp. Dassonvillei* DSM 43111	MW827875	95.06	none	White
TKL257	*Streptomyces ferrugineus* HV38	MW827889	99.42	none	White
TKL304	*Pseudarthrobacter siccitolerans* 4J27	MW827908	99.28	none	White
TKL320	*Pseudoclavibacter terrae* THG-MD12	MW827915	99.93	none	White
TKL325	*Streptomyces manganisoli* MK44	MW827917	98.81	none	White
TKL405	*Plantactinospora endophytica* YIM 68255	MW827936	99.41	none	White
TKL443	*Diaminobutyricimonas aerilata* 6408J-67	MW827945	98.15	none	White
TKL451	*Auraticoccus monumenti* MON 2.2	MW827948	99.56	none	White
TKL458	*Blastococcus xanthinilyticus* BMG 862	MW827951	98.72	none	White
TKL483	*Nocardioides furvisabuli* SBS-26	MW827957	99.13	none	White
TKL600	*Pseudarthrobacter phenanthrenivorans* Sphe3	MW827991	99.34	none	White
TKL647	*Kineococcus radiotolerans* SRS30216	MW828009	97.72	none	White
TKL651	*Streptomyces griseoviridis* NBRC 12874	MW828013	98.82	none	Red
TKL676	*Cellulosimicrobium marinum* RS-7-4	MW828022	99.20	none	White
TKL727	*Arthrobacter agilis* DSM 20550	MW828030	99.56	none	White
TKL860	*Citricoccus zhacaiensis* FS24	MW828042	98.50	none	White
TKL920	*Neomicrococcus lactis* DW152	MW828058	100.00	none	White
TKL955	*Mycolicibacterium iranicum* DSM 45541	MW828071	100.00	none	White
TKL1023	*Streptomyces griseoviridis* NBRC 12874	MW828083	98.98	none	White
TKL1045	*Micrococcus flavus* LW4	MW828091	99.93	none	White
TKL1080	*Rothia mucilaginosa* ATCC 25296	MW828107	99.50	none	White
TKL1151	*Blastococcus aggregatus* DSM 4725	MW828137	99.49	none	White
TKL1164	*Micrococcus aloeverae* AE-6	MW828144	98.69	none	Orange
TKL391	*Pseudochrobactrum saccharolyticum* CCUG 33852	MW827930	100.00	G36	White
TKL392	*Pseudochrobactrum saccharolyticum* CCUG 33852	MW827931	100.00	G36	White
TKL452	*Pseudochrobactrum saccharolyticum* CCUG 33852	MW827949	100.00	G36	White
TKL856	*Psychrobacter pulmonis* CECT 5989	MW828039	99.85	G36	White
TKL883	*Pseudomonas songnenensis* NEAU-ST5-5	MW828049	98.65	G36	White

Strain No.	Similarity Strain	Accession No.	Similarity/ %	Group	Color
TKL925	*Lysobacter bugurensis* ZLD-29	MW828059	98.96	G36	White
TKL290	*Chelativorans multitrophicus* DSM 9103	MW827899	98.82	G37	White
TKL439	*Chelativorans multitrophicus* DSM 9103	MW827943	98.44	G37	White
TKL626	*Pantoea eucrina* LMG 2781	MW828002	99.93	G38	White
TKL823	*Paracoccus aerius* 11410	MW828036	98.51	G38	Red
TKL866	*Pseudomonas songnenensis* NEAU-ST5-5	MW828045	99.36	G38	White
TKL448	*Altererythrobacter soli* MN-1	MW827946	97.86	G39	White
TKL675	*Altererythrobacter soli* MN-1	MW828021	98.00	G39	White
TKL500	*Altererythrobacter soli* MN-1	MW827962	98.01	G40	White
TKL655	*Altererythrobacter soli* MN-1	MW828014	97.85	G40	White
TKL798	*Paracoccus hibisci* THG-T2.8	MW828033	99.23	G40	White
TKL8	*Acinetobacter lwoffii* NCTC 5866	MW827798	99.78	G41	White
TKL20	*Acinetobacter lwoffii* NCTC 5866	MW827805	99.85	G41	White
TKL1109	*Pseudomonas songnenensis* NEAU-ST5-5	MW828118	98.79	G41	White
TKL863	*Devosia chinhatensis* IPL18	MW828043	97.59	G42	White
TKL1033	*Pseudomonas songnenensis* NEAU-ST5-5	MW828087	98.79	G42	White
TKL510	*Pseudomonas songnenensis* NEAU-ST5-5	MW827965	99.00	G43	White
TKL593	*Pseudomonas xanthomarina* DSM 18231	MW827986	99.43	G43	White
TKL595	*Pseudomonas songnenensis* NEAU-ST5-5	MW827988	98.79	G43	White
TKL603	*Pseudomonas songnenensis* NEAU-ST5-5	MW827992	98.85	G43	White
TKL606	*Pseudomonas xanthomarina* DSM 18231	MW827994	99.50	G43	Gold
TKL624	*Paracoccus hibisci* THG-T2.8	MW828001	99.23	G43	White
TKL870	*Pararhodobacter aggregans* D1-19	MW750564	96.38	G43	White
TKL917	*Pseudochrobactrum saccharolyticum* CCUG 33852	MW828056	98.91	G43	White
TKL947	*Pseudomonas songnenensis* NEAU-ST5-5	MW828068	99.29	G43	Light Green
TKL957	*Pseudomonas songnenensis* NEAU-ST5-5	MW828072	99.22	G43	White
TKL997	*Pseudomonas kunmingensis* HL22-2	MW828074	99.28	G43	White
TKL1020	*Pseudomonas songnenensis* NEAU-ST5-5	MW828082	99.27	G43	White
TKL1043	*Pseudomonas songnenensis* NEAU-ST5-5	MW828089	99.28	G43	White
TKL1050	*Pseudomonas songnenensis* NEAU-ST5-5	MW828094	99.05	G43	White
TKL1066	*Pseudomonas songnenensis* NEAU-ST5-5	MW828103	99.15	G43	White
TKL1083	*Acinetobacter lwoffii* NCTC 5866	MW828108	99.64	G43	White
TKL1110	*Pseudomonas songnenensis* NEAU-ST5-5	MW828119	99.29	G43	White
TKL1118	*Psychrobacter pulmonis* CECT 5989	MW828121	99.71	G43	Green
TKL1126	*Pseudomonas xanthomarina* DSM 18231	MW828126	99.22	G43	White
TKL1145	*Pseudomonas songnenensis* NEAU-ST5-5	MW828135	99.22	G43	White
TKL1165	*Pseudomonas songnenensis* NEAU-ST5-5	MW828145	98.54	G43	White
TKL533	*Pseudomonas songnenensis* NEAU-ST5-5	MW827971	98.93	G44	White
TKL948	*Pseudomonas songnenensis* NEAU-ST5-5	MW828069	98.82	G44	White
TKL1028	*Altererythrobacter soli* MN-1	MW828086	97.39	G44	White
TKL1086	*Pseudomonas songnenensis* NEAU-ST5-5	MW828109	99.22	G44	White
TKL35	*Altererythrobacter soli* MN-1	MW827814	97.95	none	Gold
TKL166	*Massilia agri* K-3-1	MW827866	98.85	none	White

Strain No.	Similarity Strain	Accession No.	Similarity/ %	Group	Color
TKL478	*Rhizobium halotolerans* AB21	MW827955	98.07	none	White
TKL642	*Altererythrobacter soli* MN-1	MW828007	98.00	none	White
TKL855	*Pseudochrobactrum saccharolyticum* CCUG 33852	MW828038	99.85	none	Pink
TKL865	*Paracoccus hibisci* THG-T2.8	MW828044	99.16	none	Red
TKL876	*Pseudochrobactrum saccharolyticum* CCUG 33852	MW828048	99.78	none	White
TKL930	*Shigella flexneri* ATCC 29903	MW828061	99.86	none	White
TKL936	*Pseudomonas songnenensis* NEAU-ST5-5	MW828063	99.29	none	Yellow
TKL1034	*Pseudomonas songnenensis* NEAU-ST5-5	MW828088	99.27	none	White
TKL497	*Pontibacter rugosus* KYW1030	MW827961	97.65	none	White
TKL664	*Sphingobacterium kitahiroshimense* 10C	MW828018	99.56	none	White
